# Structural basis for Glycan-receptor binding by mumps virus hemagglutinin-neuraminidase

**DOI:** 10.1038/s41598-020-58559-6

**Published:** 2020-01-31

**Authors:** Rosa Ester Forgione, Cristina Di Carluccio, Marie Kubota, Yoshiyuki Manabe, Koichi Fukase, Antonio Molinaro, Takao Hashiguchi, Roberta Marchetti, Alba Silipo

**Affiliations:** 1 Department of Chemical Sciences, Complesso Universitario Monte Sant’Angelo, University of Naples Federico II, Via Cintia 4, I-80126 Napoli, Italy; 2 Department of Virology, Faculty of Medicine, Kyushu University, Fukuoka, 812-8582 Japan; 3 Core for Medicine and Science Collaborative Research and Education, Project Research Center for Fundamental Science, Osaka University, 1-1 Machikaneyama, Toyonaka, Osaka 560-0043 Japan

**Keywords:** Chemical biology, Organic chemistry

## Abstract

Mumps virus is one of the main cause of respiratory illnesses in humans, especially children. Among the viral surface glycoproteins, the hemagglutinin – neuraminidase, MuV-HN, plays key roles in virus entry into host cells and infectivity, thus representing an ideal target for the design of novel inhibitors. Here we report the detailed analysis of the molecular recognition of host cell surface sialylated glycans by the viral glycoprotein MuV-HN. By a combined use of NMR, docking, molecular modelling and CORCEMA-ST, the structural features of sialoglycans/MuV-HN complexes were revealed. Evidence for a different enzyme activity toward longer and complex substrates compared to unbranched ligands was also examined by an accurate NMR kinetic analysis. Our results provide the basis for the structure-based design of effective drugs against mumps-induced diseases.

## Introduction

Mumps virus (MuV) is an aerosol-transmitted human pathogen belonging to the genus *Orthorubulavirus* of the family *Paramyxoviridae* that is composed by enveloped, non-segmented, negative-strand RNA viruses^1,2^. Paramyxoviruses also include avian orthoavulavirus 1 (newcastle disease virus), murine respirovirus (sendai virus), mammalian orthorubulavirus 5 (parainfluenza virus 5), measles morbillivirus and nipah henipavirus. MuV causes a systemic viral illness characterized by painful swelling of the salivary parotid glands. In acute phase, MuV infection can affect other tissues and organs, resulting in a wide array of inflammatory reactions, including orchitis, myocarditis, pancreatitis and nephritis^3^. In rare cases, the infection can also spread to the central nervous system (CNS), leading to meningitis and encephalitis.

According to the WHO^4^, the MuVs strains are classified into 12 genotypes, designated as A to N, based on nucleotide sequence analysis^5,6^ and containing genes encoding for nucleocapsid (N), phospho (P), matrix (M), fusion (F), small hydrophobic (SH), hemagglutinin-neuraminidase (HN) and large (L) proteins^7,8^. Each of these proteins plays a crucial role for virus entry, replication, assembly and budding. Briefly, the N, P and L proteins are located inside MuV virion and account for genome transcription and replication. The M protein is important for virion assembly, reproduction and also in the regulation of the transcription and replication phenomena. The SH protein is implicated in the evasion of the host immune response^9^. Both HN and F glycoproteins, located on the viral envelope, are responsible for adhesion and fusion to the target cells^10–12^ and represent the major targets of neutralizing Abs^13^. As for the majority of paramyxoviruses, HN protein from mump virus (MuV-HN) exhibits both hemagglutinin and neuraminidase activity, playing a fundamental role in the membrane fusion process during the virus entry as well as in the release and spread of the virus^14^.

The *N*-acetyl neuraminic acid is one of the most common sialic acids (Sia), which typically exists as a non-reducing terminal component of many glycoconjugates expressed on the human cells surface, representing a usual structural element of the mammalian glycome and playing pivotal roles in many physiopathological processes^15^. Its role in mediating bacterial and viral infections^16^ is essential and is indeed exploited by many pathogens, including paramyxoviruses^17–19^, for entering the host cells. As mentioned above, the MuV-HN plays different functions: it recognizes and binds sialic acid containing structures, including glycosphingolipids, *N*- and *O*-glycans exposed on host cell surface^18,20^; promotes the fusion activity of the F protein, leading the fusion of MuV envelope and the host cell membrane at the cell surface; finally, cleaves the sialic acid residues from glycan receptors and progeny virus particles. The neuraminidase activity of MuV-HN has important effect on the virus budding, as prevents the viral self-agglutination and facilitates the release of the virus from the host cell surface^18,21^.

Given the important roles of MuV-HN in the infectivity of the virus, the impairment of its function represents an ideal target to impede either the viral infection and spreading, by hindering the virus attachment to host cell, the promotion of fusion activity and the neuraminidase function. We previously determined the crystal structure of the MuV-HN, showing its preference for α-2,3-linked sialic acid in linear chains as glycan-receptors; in addition, the co-crystals with 3′-sialyllactose revealed key interactions with a long saccharide chain. Here, with the aim to further describe the dynamic events of recognition and binding processes triggering MuV infectivity, the structural basis of sialylated ligands binding to MuV-HN were further investigated by a combination of NMR techniques and computational studies. In addition, the kinetic parameters of the sialoglycans hydrolysis catalyzed by the neuraminidase activity of HN were evaluated. Our results provide further insights into the molecular mechanisms of MuV infection, representing a step beyond into the development of more effective antiviral vaccines.

## Results

Given the ability of MuV-HN to recognize and cleave α-2,3-linked sialoglycans on host cell surface, we chose to investigate the binding by MuV-HN to different substrates (Scheme S1), representative of glycosphingolipids, *O*-linked glycoproteins, as well as of *N*-linked glycans.

### Kinetic analysis of the hydrolysis of 1 and 2 by MuV-HN

**Figure 1:**
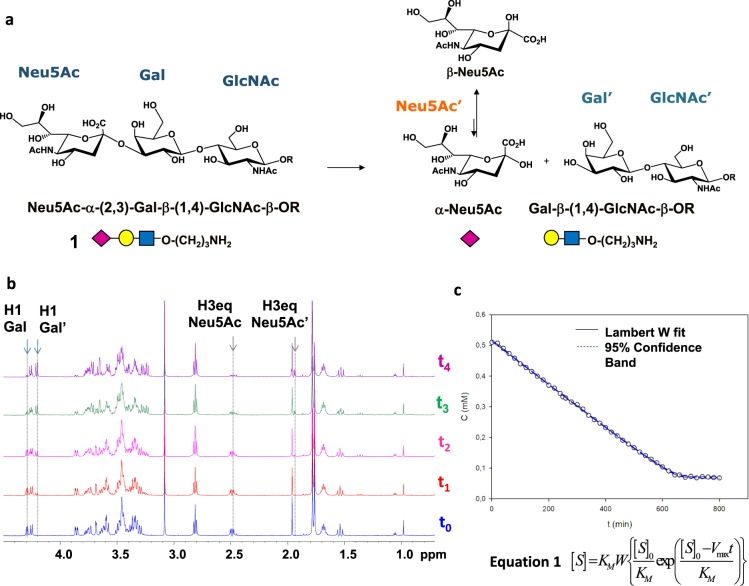
(**a**) Scheme of the mechanism of hydrolysis of substrate **1** catalyzed by MuV-HN neuraminidase. The 3D Symbol Nomenclature For Glycans (SNFG) has been used. Chem Draw 2006 Software (http://www.cambridgesoft.com) was used to draw the sugar moieties. (**b)** Section of ^1^H-NMR spectra at different time of the enzymatic reaction have been reported. 7.5 μM of MuV-HN and 550 μM of **1** were solved in PBS deuterate buffer (pH = 7). The NMR quantification of the substrate concentration was performed by integration of the well-dispersed resonances of **1** (H1 Gal, H3_eq_ Neu5Ac). The NMR spectra were plotted by using the xwinplot tool of TopSpin 3.6.1 software (www.bruker.com). (**c)** Analysis of the MuV-HN kinetics toward substrate **1** by means of the explicit reformulation of the integrated form of MM equation with Lambert W fit as solution. The plot of the concentration of **1** as function of time has been reported. The substrate concentration was evaluated from the H3_eq_ resonance of Sia unit. The fit of the kinetic data by the Lambert-W fit afforded a K_M_ value of 12 μM and V_max_ of 7 * 10^−3^ (mM/min). The blue dashed line represented the confidence interval of the fit. Data were analyzed by Sigma Plot 11.0 (http://www.sigmaplot.co.uk/).

As first step to get into the structural details of binding, we examined the kinetic mechanism of MuV-HN, i.e., the enzyme kinetic parameters were evaluated by progress curve analysis using NMR detection of products and substrates hydrolysis of **1** and **2** (Scheme S1, Fig. 1)^22^.

Thus, the hydrolysis of the sialylated trisaccharide **1** (Fig. 1) was followed by ^1^H NMR; as shown in the 1D NMR spectra reported in Fig. 1, MuV–HN cleaved the terminal Sia moiety, leaving *N*-Acetyllactosammine (LacNAc) and a residue of reducing sialic acid (*red-*Neu5Ac). As shown in Fig. 1b, the progress of the hydrolysis was evident from the decrease of the signals of the substrate (as H1 Gal and H3_eq_ Neu5Ac) and the simultaneous increase of the intensity of the products resonances (as H1 Gal’ and H3_eq_ Neu5Ac’). As expected, the hydrolysis promoted by MuV-HN proceeded with a retaining mechanism, involving the net retention of configuration of the cleaved sugar^23,24^ (Figs. 1a and S1); the successive and progressive anomerization process then caused the decrease of the α-NeuAc anomer in favor of the most stable β-Neu5Ac anomer.

In order to derive the kinetic parameters of the hydrolysis, selected isolated NMR resonances (as the signal of H3_eq_ of Neu5Ac unit in the substrate) were integrated and subsequently plotted as function of time. The variation of the substrate concentration with time can be efficiently described by the explicit reformulation of the Michaelis–Menten kinetics^25^ that provides the Lambert-W function as solution, developed by Goličnik and reported in Fig. 1c (eq. 1)^26,27^.

Thus, by using a single initial substrate concentration with no need of fluorescent probing and by analyzing the kinetic data through a nonlinear least-square fit routine, it was possible to construct the kinetic curve showed in Fig. 1c and to determine K_M_ and V_max_ values. The fit of the experimental data, whose noticeable quality corresponded to a χ^2^ value of 0.9998, provided the kinetic parameters summarized in Table 1, including a K_M_ of 12 μM and a V_max_ of 7 *10^−3^mM/min.

**Table 1:** Kinetic parameters obtained from the progress curve analysis for the hydrolysis of substrates **1** and **2** promoted by MuV-HN.

	K_M_ (µM)	V_max_ (mM/min)	Kcat (min^−1^)	Kcat/K_M_ (mM^−1^min^−1^)
trisaccharide **1**	12	0.0007	0.1	8.55
undecasaccharide **2**	3.5	0.000023	0.0025	0.65

The kinetic analysis carried out by using as substrate the longer sialylated *N*-glycan **2** (Scheme S1) supported the retaining mechanism but revealed a different kinetic activity of the enzyme toward the two different substrates, **1** and **2** (Table 1, Figs. 1 and S2). As expected, the ^1^H NMR spectra revealed the initial production of α-Neu5Ac after incubation of **2** with MuV-HN, then followed by its anomerization (Fig. S2b). However, the comparison of the kinetic parameters calculated for the hydrolysis of **1** and **2** (Table 1) clearly showed that the receptor was more active toward **1**, as suggested by the 12-fold higher value of the kinetic efficiency^28^ (K_cat_/K_M_) (see below for further discussion).

### Molecular recognition of sialoglycans by MuV-HN protein

The interaction between MuV-HN and different sialo-glycans was characterized by means of ligand-based NMR techniques, including saturation transfer difference (STD) NMR^29–31^ and transferred-NOESY (tr-NOESY)^30,32^. Given the neuraminidase activity of MuV-HN, the NMR experiments were performed at 283 K to slow down the kinetic of sialidase activity and to hamper the hydrolysis of the substrate during the acquisition time.

**Figure 2:**
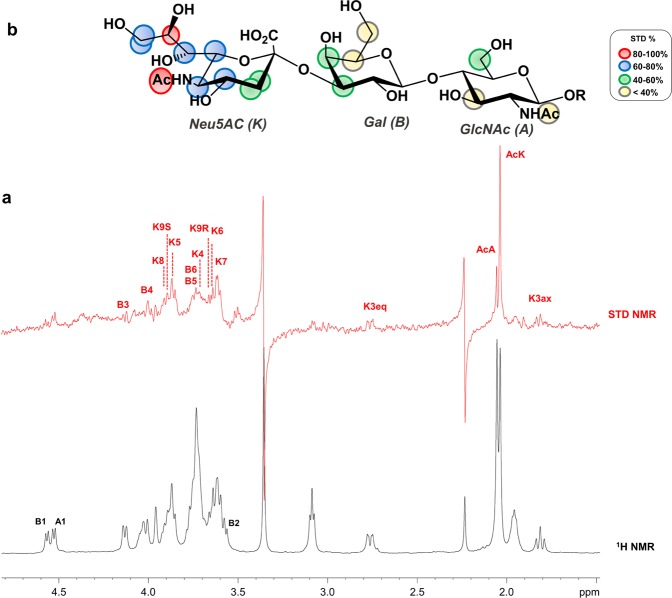
STD NMR analysis of trisaccharide **1** in the interaction with MuV-HN. (**a)** The off-resonance spectrum as reference (black) and the STD (red) of MuV-HN–trisaccharide-**1** mixture with a molecular ratio of 1:70, at 283 K. (**b)** Epitope map of **1**. STD percentages were calculated by (I_0_ – I_sat_)/I_0_ ratio_,_ normalized with respect to the highest STD signal of the acetyl group belonging to sialic acid. Chem Draw 2006 Software (http://www.cambridgesoft.com) was used to draw the substrate. The NMR spectra were plotted by using the xwinplot tool of TopSpin 3.6.1 software (www.bruker.com).

The STD NMR analysis of the trisaccharide **1** was used to map the glycan receptor interacting epitope, namely, to identify the moieties deeply involved in the interaction with MuV-HN (Fig. 2). By comparing off-resonance and STD NMR spectra (Fig. 2a), changes in the intensity and multiplicity of specific signals were observed. As expected, the sialic acid residue (**K**) was the portion of **1** mostly involved in the interaction with MuV-HN, with the highest STD effect belonging to the *N*-acetyl group and strong involvement in the interaction also detected for H-8 **K**, (saturation transfer above 90%); also, protons H-4, H-5, H-6, H-7 and H-9, exhibited a considerable participation to the binding process (above 70%). Lower STD signals were observed for the H-3 axial and equatorial protons of **K**. These data confirmed the key role of the sialic acid residue in the interaction process, likewise a significant transfer of magnetization from MuV-HN to **1** was also observed for the galactose unit (**B**). In particular, its H-3 and H-4 proton signals exhibited STD enhancements above 50%. Finally, proton signals H-3, H-6 and the *N*-acetyl group of **A** gave also rise to STD enhancements, although to a lesser extent, further suggesting that the *N*-acetylglucosamine unit pointed further away from the binding pocket of MuV-HN than **K** and **B** units. The different involvement of the saccharide units of **1** in the recognition and binding process was also confirmed by the significant difference in terms of STD intensity of the two singlets around 1.8 ppm belonging to the acetyl groups of the *N*-acetylglucosamine (**A**) and sialic acid (**K**) residues (Fig. 2a). Thus, from the STD NMR analysis it was possible to map the interacting epitope of the trisaccharide **1** (Fig. 2b) with MuV-HN, able to differently accommodate not only the Sia-Gal units, which made the most relevant contacts with the protein, but also the adjacent *N*-acetylglucosamine unit, which contributed to the recognition process.

**Figure 3:**
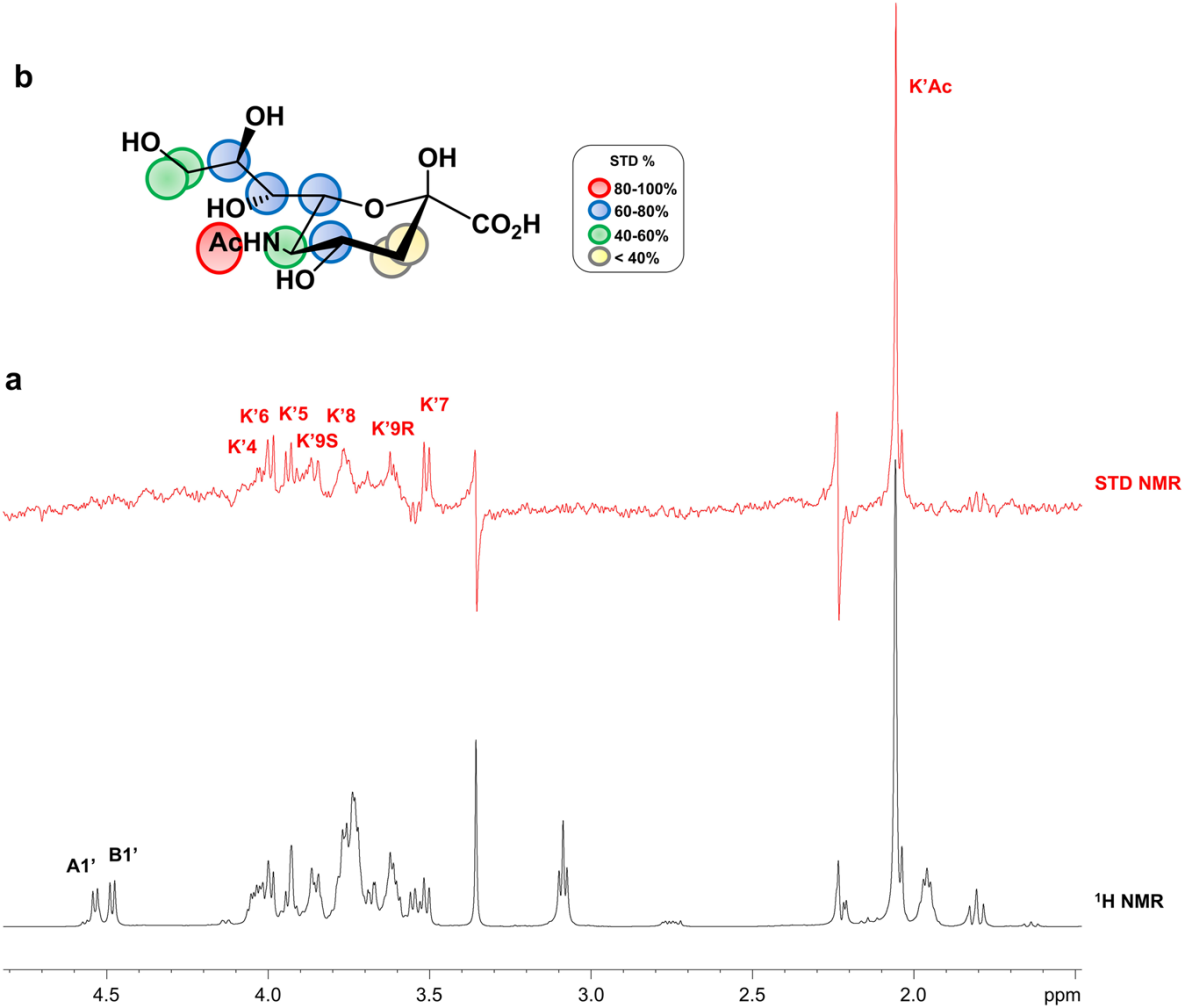
STD NMR analysis of reducing Sia produced during the hydrolysis of **1** by MuV-HN. (**a)** The off-resonance spectrum as reference (black) and the STD (red) of MuV-HN–trisaccharide-**1** mixture upon hydrolysis reaction. (**b)** Epitope map of reducing Sia. STD percentages were calculated by (I_0_ – I_sat_)/ I_0_ ratio_,_ normalized with respect to the highest STD signal of the acetyl group belonging to sialic acid. Chem Draw 2006 Software (http://www.cambridgesoft.com) was used to draw the sugar. The NMR spectra were plotted by using the xwinplot tool of TopSpin 3.6.1 software (www.bruker.com).

Interestingly, we found that the protein was able to bind the reducing sialic acid produced during the hydrolysis reaction. Indeed, STD NMR analysis conducted on the reaction mixture upon the hydrolysis of the substrate **1** (Fig. 3) clearly showed STD signals belonging to the more stable β-anomeric form (β-Sia) of the reducing Sia, as further confirmed by studying the interaction between free reducing sialic acid and the MuV-HN (Fig. S3).

We could also demonstrate the specificity of the hydrolysis by MuV-HN, as the substrate **3**, containing α-2,6-linked sialic acid, was neither recognized nor hydrolyzed by MuV-HN (see Fig. S4, the lack of STD signals is diagnostic of absence of interaction).

The ability of MuV-HN to recognize and interact with different cell surface sialylated glycoconjugates was then confirmed by investigating the interaction of MuV-HN with a longer sialoglycan, the undecasaccharide **2** (Scheme S1, Fig. S5), a canonical sialylated complex type glycan exposed on mammalian cells. In accordance with the epitope map of **1**, the STD analysis of **2** revealed that the sialic acid moiety received the largest fraction of saturation transfer. STD enhancements were also observed for protons belonging to galactose and *N*-acetyglucosamine residues, while no STD contributions came from the other sugar units, thus suggesting that the accommodation of complex *N*-glycans in the MuV-HN binding site is not influenced by the length of the glycan chain. Nevertheless, the lower enzymatic activity of the enzyme toward the substrate **2** showed by the results of the kinetic analysis (see above) highlighted how the recognition, accommodation and subsequent enzymatic hydrolysis was partially hampered by the steric hindrance of the glycan chains (see below and the discussion).

**Figure 4:**
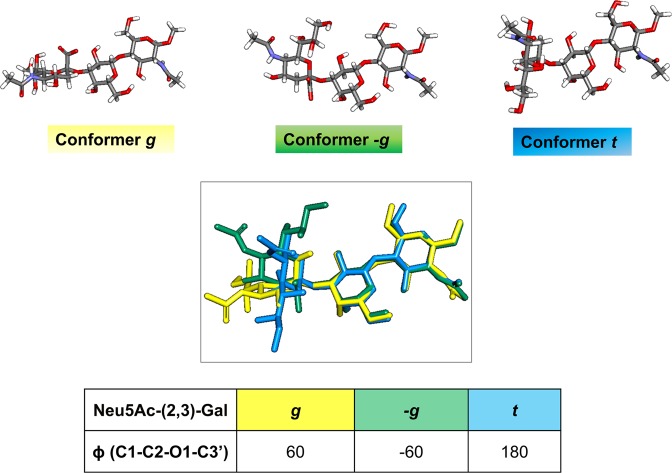
Conformational behaviour of substrate **1**. The three major conformations in solution of the **1** that differ from the value of the phi torsion of the Siaα2-3Gal linkage: the g, -g and t. The glycosidic torsion angles definition was ϕ = C1-C2-O2-C3′, ψ = C2-O2-C3′-H3′. Discovery Studio 2016 Software (www.3dsbiovia.com) was used to draw the conformations.

Further details on sialoglycans recognition by MuV-HN were gathered by tr-NOE analysis, which allowed to derive the bioactive conformation of **1**. First, the combined use of NMR and Molecular Mechanic simulations revealed that, in the free state, the substrate **1** exhibited an equilibrium between different conformational states, mainly described by the so known –*g*, *g, t* conformers and differing by the ϕ (H1-C1-O-CX’) torsion angle around the Sia–α–(2,3)-Gal glycosidic linkage (−60°/60°/180° respectively)^33^ (Fig. 4). The assessment of the conformational behavior of **1** was performed by analyzing NOE and tr-NOE contacts obtained in 2D NOESY spectra acquired both in free and in bound states (Table 2, see also Supplementary Information, Fig. S6). Among the others, the key distances between the H-3 methylene protons of the sialic acid and H-3 and H-4 protons of the galactose residue suggested that substrate **1** exhibited a preference for the *t* conformer in the bound state (Figs. 4 and S6).

**Table 2:** Conformational analysis of trisaccharide **1** in the free and bound states compared with respect to the conformer populations described by the ϕ torsion angle values, according to the literature^58^.

Distance	Conformer				
	*−g* Φ = −60° Ψ = 0°	+*g* Φ = 60° Ψ = 0°	*t* Φ = 180° Ψ = 0°	Exp Free State	Exp. Bound State
H3 Gal-H3ax Sia	4.19	3.11	2.18	2.74	2.35
H3 Gal-H3eq Sia	4.20	2.03	3.40	3.36	3.43
H4 Gal-H3eq Sia	4.15	2.25	5.15	4.24	Not observed
H4 Gal-H3ax Sia	5.04	2.13	4.45	3.94	4.31

**Figure 5:**
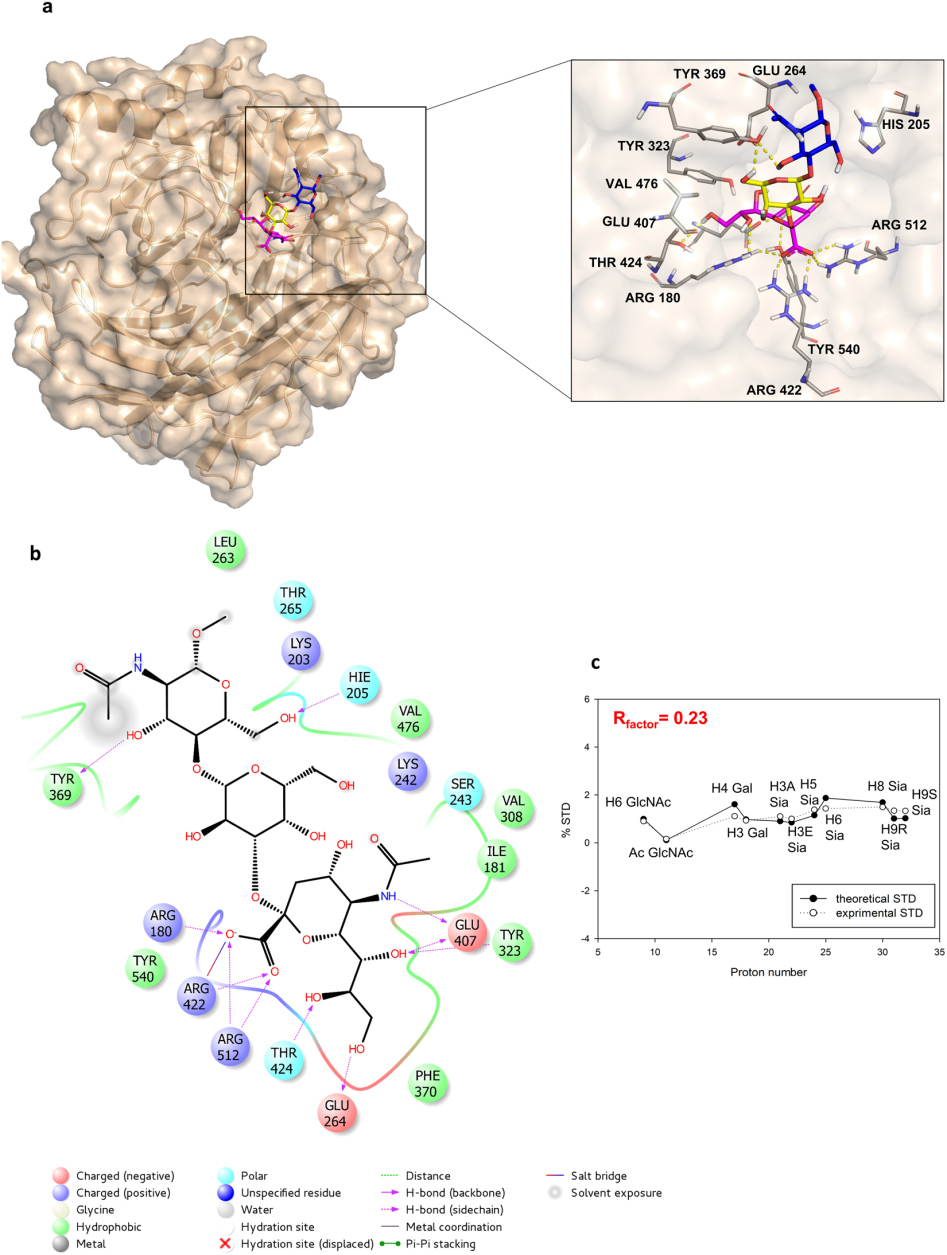
3D model of MuV-HN in complex with trisaccharide **1** and CORCEMA analysis. **(a**) Close up view of ligand **1** binding mode at the MuV-HN active site, drawn by using Pymol 2.3 Software (https://pymol.org/2/). The main amino acid residues involved in the binding are represented in stick. Galactose and *N*-acetyl Glucosamine residues are depicted in yellow and blue, respectively. (**b)** Two-dimensional plot illustrating the interactions of the sialylated trisaccharide **1** with the residues in the binding pocket of MuV-HN derived by a tool included in Maestro Version 10.5.014 (https://www.schrodinger.com/maestro). Dotted arrows represent hydrogen bonds with functional groups from side chains and solid arrows such with functional groups of the backbone. The residues shown, close to the ligand, are involved into hydrophobic and polar interactions. (**c)** Comparison between experimental (dashed line) and theoretical (solid line) STD data for the selected 3D complex of the interaction between trisaccharide **1** and MuV-HN head domain.

These data were further confirmed by applying a combined approach based on computational studies and the use of CORCEMA-ST^34,35^ (Complete relaxation and conformational exchange matrix analysis of saturation transfer) program that, starting from the 3D coordinates of a given protein-ligand complex, allows the prediction of the STD effects for each ligand proton (Fig. 5). First, docking calculations of the MuV-HN− **1** complex were carried out using the recently published crystal structure (PDB ID, 5b2c) of MuV-HN receptor-binding head domain (Fig. 5a)^18^. The substrate **1** was built and optimized by means of Maestro suite of programs^36,37^. All the three main conformers, namely -*g*, *g*, *t*, were considered for computational 3-D structure calculations. The docking analysis highlighted that, for each conformer, a consistent binding mode with good theoretical energy was possible (Fig. S7, Table S1). Thus, three representative complexes were submitted to CORCEMA-ST program to compare calculated and experimental STD effects for each conformer. The parameter that indicates the quality of the fit is a normalized root-means square deviation (RMSD) value, called R-NOE factor (Figs. 5 and S8). Among the three examined structures, the complex built with the *t* conformer (Fig. 5) exhibited the best agreement between theoretical and experimental STD data (R-NOE of 0.23, Figs. 5c and S8) thus confirming that the ligand preferentially adopted a *t* conformation around Sia-Gal linkage in the bound state. From the CORCEMA-ST prediction, protons from all the substrate units exhibited STD effects, thus assessing their involvement in the interaction with MuV-HN. In detail, the highest predicted STD effects belonged to the Acetyl group of Sia, due to the strong hydrogen bond between the *N*-Acetamide moiety of Sia and the Glu407 backbone, together with hydrophobic contacts involving the acetyl group located in a hydrophobic cleft constituted by Val and Ile residues. Also, H-8 proton of Sia unit exhibited more than 80% of saturation, in agreement with the contacts occurring between the hydroxyl group in position 8 and Tyr323 and Thr424 residues of MuV-HN (Fig. 5a,b). Significant STD effects were also predicted for H-5, H-6, H-7, H-9 of Sia, all involved in interactions with Tyr323 and Glu264 residues. Lower STD enhancements were instead predicted for H-3 eq and H-3 ax of Sia and H-3, H-4, H-5, H-6 of Gal in accordance with their proximity to the receptor surface and the weaker hydrophobic contacts observed in the complex. On the other hand, no theoretical STD NMR effects arose for H-1 and H-2 protons of Gal, as they pointed outside the glycan-receptor binding pocket. Finally, STD enhancements were predicted for the *N*-acetamide moiety and the protons H-2, H-4, H-6, all belonging to the GlcNAc face that was straight directed toward the MuV-HN surface.

It is worth to note that the profiles of the predicted STD effects, belonging to the other two possible conformers (*-g* and *g*, see Figs. 4, S7 and S8 and above), were very far from the corresponding experimental STD enhancements and for that, resulted in higher R-NOE values (Fig. S8). Therefore, CORCEMA-ST analysis allowed to further exclude that trisaccharide **1** adopted these conformations when interacting with MuV-HN.

Overall, the combination of NMR, docking and CORCEMA analysis permitted to draw an accurate model of the interaction between trisaccharide **1** and the MuV-HN head domain (Fig. 5a) and select the right conformer among three different possible available. According to our model, the *t* conformer of **1** selected in the bound state was able to fully fit the binding pocket of the MuV-HN, characterized by a rather extended topology, which allowed establishing several interactions with almost the entire sugar backbone of **1**, as clearly shown in the 2D representation depicted in Fig. 5b. In the final selected MuV-HN/**1** model, showing the best agreement with the STD and NOE-based experimental data, the entire trisaccharide **1** stretched in the binding pocket of the MuV-HN, establishing several polar contacts with the active site residues which are conserved among all MuV genotypes. The major determinant of the binding was the sialic acid moiety that established electrostatic interactions and hydrogen bonds with catalytic site MuV-HN polar residues mainly through its carboxylate, glycerol chain and *N*-acetamide group. The strong ionic interactions formed by the Sia carboxylate group and the guanidinium groups of Arg180, Arg422 and Arg512 of the MuV-HN were essential for the binding^38^. The Sia unit was further involved in hydrogen bonds through the hydroxyl groups of its glycerol chain and Tyr323, Glu264 residues of the MuV-HN, together with the interaction between its *N*-Acetamide moiety and Glu407 residue. Also, the acetyl group of Sia contributed to the binding, being engaged in hydrophobic interactions with the receptor non-polar residues. Even the Gal moiety revealed to be in close contact with the protein, though stabilized by weaker interactions with the MuV-HN surface, in detail by the contacts involving Val476 and Tyr369 residues of the binding cavity of MuV-HN. Finally, the third sugar unit from the reducing end mainly contributed to the overall interaction with the MuV-HN highly conserved Tyr369 and His205 residues, though displayed a lower participation to the recognition and binding processes. These results detailed the previously underlined^18,20^ contribution of the third sugar unit from the reducing terminal in the interaction with MuV-HN, together with the crucial role of Tyr369 in the recognition of sialylated glycan receptors.

**Figure 6:**
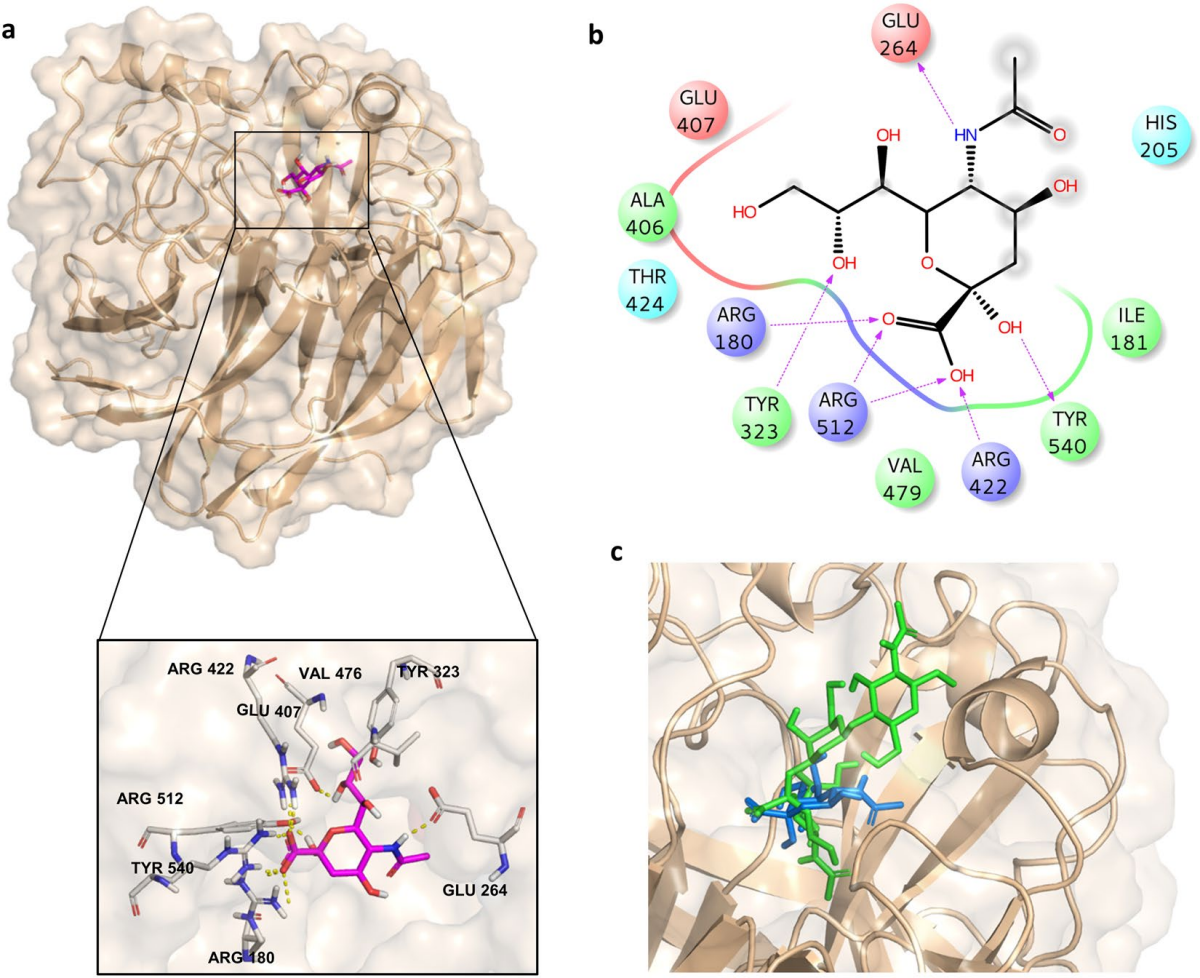
3D model of MuV-HN in complex with β-Sia. **(a)** Close up view of β-sia binding mode at the MuV-HN active site, drawn by using Pymol 2.3 Software (https://pymol.org/2/). The main amino acid residues involved in the binding are represented in stick. (**b)** Two-dimensional plot illustrating the interactions of the β-Sia with the residues in the binding pocket of MuV-HN derived by a tool included in Maestro Version 10.5.014 (https://www.schrodinger.com/maestro). Dotted arrows represent hydrogen bonds with functional groups from side chains and solid arrows such with functional groups of the backbone. The residues shown, close to the ligand, are involved into hydrophobic and polar interactions. Although the different orientation inside MuV-HN binding site of the β-Sia with respect to the α-anomer in **1**, the triad of arginine residues of the MuV-HN binding pocket was again engaged in strong ionic interactions with the Sia’s carboxylate. (**c)** Superimposition of β-Sia blue and trisaccharide **1** green in the MuV-HN binding site.

Interestingly, the *red*-β–Sia displayed a different orientation inside the receptor pocket with respect to the Sia unit of **1** (Fig. 6), establishing strong ionic interactions between its carboxylic group and the highly conserved active site arginine triad, but resulting in a slightly different set of interactions involving its lateral chain and *N*-Acetamide moiety. Particularly, the *N*-Acetyl group of Sia formed a hydrogen bond with Glu264 residue in place of Glu407, whereas the lateral chain engaged polar interactions mainly with Tyr323 and Glu407 residues. Furthermore, the hydroxyl group in position **2** of the sugar established a hydrogen bond with the Tyr540 residue.

**Figure 7:**
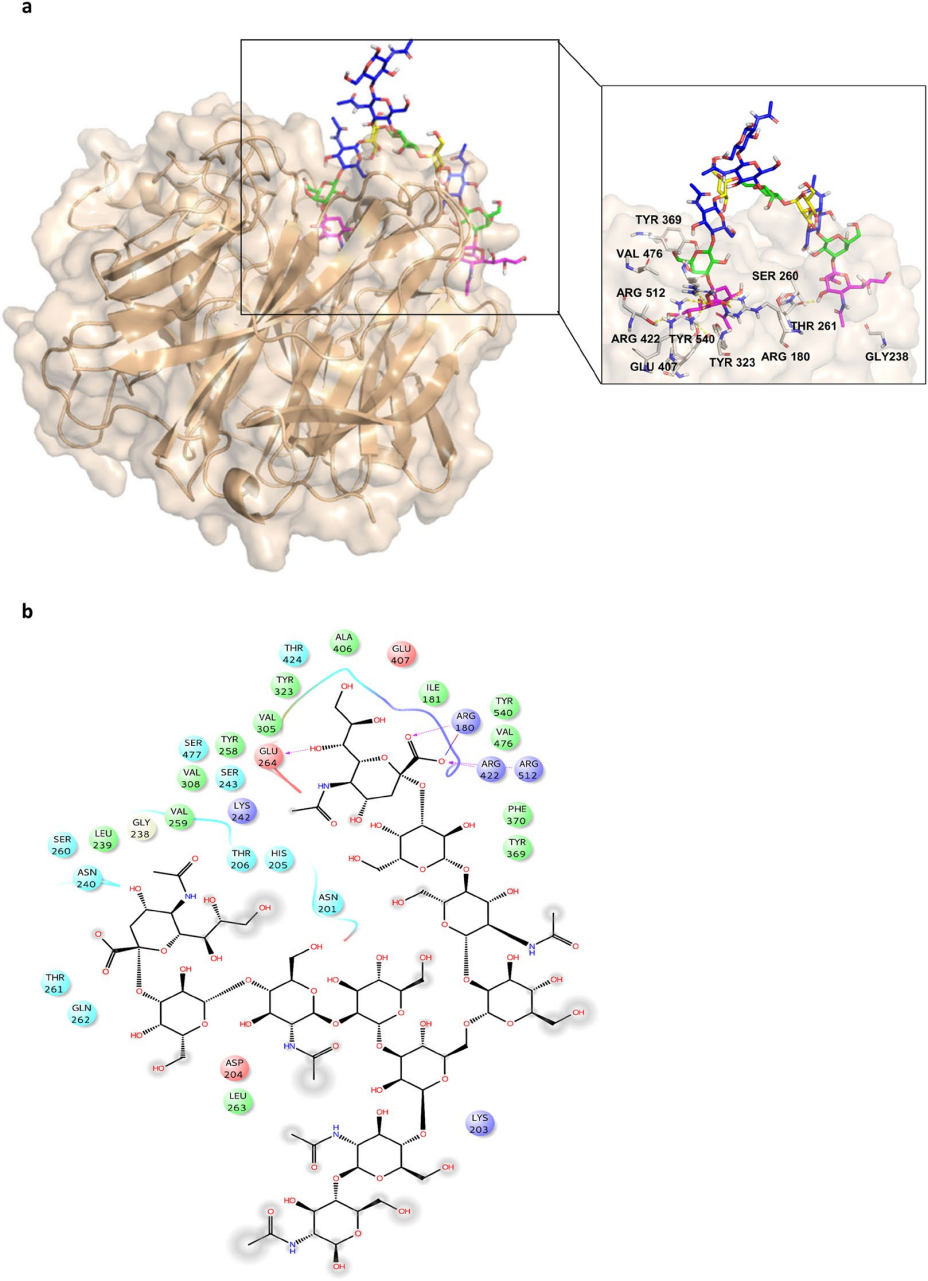
3D model of MuV-HN in complex with undecasaccharide **2**. **(a)** Close up view of ligand **2** binding mode at the MuV-HN active site, drawn by using Pymol 2.3 Software (https://pymol.org/2/). The main amino acid residues involved in the binding are represented in stick. Galactose and *N*-acetyl Glucosamine residues are depicted in yellow and blue, respectively. (**b)** Two-dimensional plot illustrating the interactions of the sialylated undecasaccharide **2** with the residues in the binding pocket of MuV-HN derived by a tool included in Maestro Version 10.5.014 (https://www.schrodinger.com/maestro). Dotted arrows represent hydrogen bonds with functional groups from side chains and solid arrows such with functional groups of the backbone. The residues shown, close to the ligand, are involved into hydrophobic and polar interactions.

On the other hand, docking calculations highlighted that the pattern of interactions established in the undecasaccharide **2**/HN complex was comparable to that of the trisaccaride **1/**HN (Fig. 5); the triad of arginine residues of the MuV-HN binding pocket was again engaged in strong ionic interactions with the Sia’s carboxylate; the Sia’s lateral chain established hydrogen bonds with the polar residues of the receptor, however, a reduced polar network was observed (Fig. 7).

MuV-HN was indeed able to accommodate either the α–1,6 or α–1,3 arm of the sialylated undecasaccharide in its binding pocket and, in both cases, the highly conserved electrostatic interactions involving the Sia carboxylate and the Arg180, Arg422, Arg512 residues were observed. However, the MuV-HN/**2** complex did not show the hydrogen bonds formed by Glu407 and Tyr323 with Sia unit, along with the interactions between the third sugar unit and Tyr369 residue. This was likely due to the higher steric hindrance given by the length and branching of **2**, that hindered the optimal accommodation of its terminal moiety into the binding pocket of the protein. This is also in accordance with previous glycan array experiments which revealed a lower affinity of MuV-HN for branched complex-type branched glycan receptors^18^. Interestingly, the sialic acid unit at the terminus of the glycan antenna that is not accommodated into the binding pocket engaged polar interactions with the MuV-HN outer surface residues (Gly238, Ser260, Thr260). These non-specific interactions could account for the higher K_M_ found with the NMR kinetic analysis of the undecasaccharide **2** with respect to the trisaccharide **1**.

## Discussion

MuV is one of the principal causes of respiratory diseases in humans, especially children. With the development of live attenuated vaccines, the prevalence of MuV infections has been dramatically reduced^5,39^; however, in the last decade, several outbreaks of MuV infection occurred worldwide, even in highly vaccinated populations^40–42^. The reasons of such resistance still need to be clarified, although the mechanism is believed either the weaning of the immunity (secondary vaccine failure) or the vaccine escape allowed by antigenic differences between the vaccine and outbreak strains^43–45^. It is worth to note that the antigenic drift mainly affects the SH and HN protein coding regions^46,47^.

Among the viral surface glycoproteins, the HN protein represents the main antigen inducing neutralizing antibodies and plays key roles for the tropism of mump viruses. The MuV-HN neuraminidase belongs to the exo-α-sialidase enzyme class (EC number 3.2.1.18) which includes enzymes able to catalyze the hydrolysis of α-(2 → 3)-, α-(2 → 6)-, α-(2 → 8)- linked sialic acid residues^48^. Upon recognition of sialylated-glycan structures of host cell surface glycolipids and glycoproteins, particularly abundant in the mucus covering the respiratory epithelia, MuV-HN induces the structural change of the adjacent MuV-F, promotes the fusion activity and also serves to remove sialic acids from the virus-budding cell surface and progeny viral particles. This neuraminidase activity is crucial to avoid the viral tethering on cell surface, the nonspecific binding onto the cell matrix components and the viral self-agglutination.

Thus, MuV-HN is an attractive target for the design of effective drugs against the diseases caused by mumps virus. Indeed, the purpose of this study was exactly to contribute to this aim by dissecting, at molecular level and in solution state, the basis of sialoglycans recognition, by establishing the proper glycan conformation selected upon binding and by describing its hydrolysis kinetic by MuV-HN.

A detailed epitope mapping of different ligands was achieved by STD NMR, allowing the identification of the saccharide region in intimate contact with MuV-HN binding pocket. In accordance with the defined crystal structure complex of MuV-HN with 3′ sialyllactose we previously reported^18,20^, all the saccharide units of the sugar chain participated in the binding with the HN protein, which was able to accommodate in its binding pocket the β-anomer of the Sia residue as well. Therefore, it was possible to establish that MuV-HN was characterized by a considerably extended binding site compared with other neuraminidases^17^.

Tr-NOESY experiments complemented with computational studies, including Docking and CORCEMA, allowed to investigate the conformational behavior of sialoglycans when binding the MuV-HN, highlighting a conformer selection. Overall, our results revealed the preference of the substrate **1** for the *t* conformer, characterized by ϕ/ψ torsion angles of 180°/−11° around the Sia-Gal glycosidic linkage and allowed to depict 3D models of MuV-HN–glycans complexes.

Docking calculations in the extended protein binding site allowed to establish the network of interactions which characterize the ability of MuV-HN to bind different sialoglycans, including the β-anomer of the sialic acid, but to accommodate also long saccharide epitopes. Interestingly, our data revealed indeed that MuV-HN was able to recognize the complex glycan undecasaccaride **2**, displaying interactions comparable to those of MuV-HN/**1** complex, however, the steric hindrance of the saccharide chains, due to the branching of substrate **2**, increased the difficulties of the protein in accommodating this glycan-receptor. These data were further supported by kinetic studies which allowed to evaluate the catalytic activity of MuV-HN by following the hydrolysis of the two sialoglycans in the presence of the HN protein. The derived values of K_M_ and V_max_, indeed, clearly showed a lower kinetic efficiency of the enzyme toward the substrate **2**.

In conclusion, the present study advances the knowledge about the ability of MuV-HN to specifically recognize α-2,3-linked sialic acid in Sialyl-LacNAc fragments and at the terminus of *N*-linked glycans commonly found on cell-surface glycoproteins. This study also revealed molecular details of the viral entry and release mechanism into/from host cells by biophysical characterization of glycan-receptors binding to the MuV-HN and their hydrolysis reactions. Our results may serve for the structure-based design and synthesis of sialic acid-derived compounds analogues acting as inhibitors of MuV-HN.

## Methods

### NMR experiments

NMR spectroscopic experiments were collected by a Bruker 600-MHz DRX instrument fitted with a cryo probe. NMR samples were dissolved in 50 mM deuterate phosphate buffer (NaCl 140 mM, Na_2_HPO_4_ 10 mM, KCl 3 mM, pH 7.4) and the [D4](trimethylsilyl)propionic acid, sodium salt (TSP, 10 uM) was used as internal reference to calibrate all the spectra. Data acquisition and processing were analyzed using TOPSPIN 3.2 software. The chemical shifts of the glycan ligands were assigned by ^1^H, COSY, TOCSY, NOESY and HSQC experiments. Ligands **1**, and **3** were purchased from Tokyo Chemical Industry Co., Ltd. (Product Number: N0949, N0950). Ligand **3** was provided from GlyTech, Inc.^49^.

^1^H NMR spectra were registered by using 16 k and 32 k data points. The homonuclear spectra were recorded with data sets of 4096x512 (t1 × t2) points and the data matrix processed with zero-filled in the F1 dimension up to 4096x2048 points. In order to improve the resolution, a cosine-bell function was used before Fourier transformation in both dimensions. Heteronuclear single quantum coherence (HSQC) experiments were carried out in the ^1^H-detected mode by single quantum coherence with proton decoupling in the ^13^C domain, setting data points of 2048x256. The method of States *et al*. was employed for the experiments in the phase-sensitive mode.

### STD NMR analysis

STD NMR experiments were accomplished at 283 K and 298 K with 32 k data points and zero-filled up to 64 k data points prior to processing. Gaussian shaped pulse train of 50 ms (field strength of 21 Hz) with an interpulse delay of 1 ms and an attenuation of 60 db was selectively sent to saturate the protein. STD NMR experiments on the protein without the ligand at different conditions of frequency irradiation and values of attenuation were performed to choose the optimal parameters to use in the mixture with ligands. The on-resonance pulse was set to 6.5 ppm and the off-resonance was acquired irradiating at a frequency of 40 ppm. A control of the absence of direct irradiation of the ligand was made at the same conditions to make sure the absence of artifacts in the STD analysis. The protein/ligand molar ratio was imposed as 1:70, with a concentration of the protein of 7.5 uM and a total saturation time was 2.00 s. An excitation sculpting with gradient pulses (esgp) was applied to suppress the water signal. The epitope mapping of ligand **1** was achieved by the calculation of the ratio (I_0_ – I_sat_)/I_0_, where (I_0_ – I_sat_) is the intensity of the signal in the STD NMR spectrum and I_0_ is the peak intensity referred to the unsaturated reference spectrum (off-resonance). The intensities of each proton of the ligand **1** were normalized to the highest STD signal. For the ligand **3**, STD NMR experiments were carried out at different irradiation frequencies (δ of 0 and 6 ppm) and by varying spin lock pulses (2 ms and 25 ms) to suppress the broad protein background.

### Tr-NOESY analysis

Homonuclear 2D ^1^H-^1^H NOESY experiments were performed at 283 K by using data sets of 4096x512 points and mixing times of 600 ms and 400 ms were chosen for the free and bound state, respectively. The protein: ligand ratio was set to 1:30.

### Kinetic analysis

For the analysis of the enzyme kinetic of HN, a 1:50 p/l molar ratio was used. The enzyme and the substrate were dissolved in 50 mM PBS/D_2_O buffer, pH 7.4. All of the spectra were recorded at 298 K. Before the addition of HN protein, a 1D proton spectrum with the application of composite pulses to carry out water presaturation (zgppr) was acquired with 32 transients (2 min experiment duration), to obtain the intensities corresponding to t = 0. Then, HN was added to the NMR tube and the solution was mixed quickly. The sample was then reintroduced into the magnet and after applying a short shimming routine, ^1^H NMR spectra using the aforementioned acquisition parameters were recorded at different time points for several hours. A timer was set to measure the delay between the addition of HN and the collection of the first NMR spectrum of the mixture. The delay time was incorporated into the previous calculations. FIDs were multiplied with an exponential function prior to Fourier transformation. All well dispersed resonances of substrate and product were integrated at each time point. The concentrations each time point was determined with respect to the integrals of the H-1 and H3_eq_ resonances of trisaccharide **1** and undecasaccharide **2** at t = 0, which corresponded to a concentration of 540 uM. The Progress curve for the transformation of **1** and undecasaccharide **2** were fitted in Sigma Plot with the equation adapted from reference^50^.

### Docking calculations

The 3D coordinates of MuV-HN head domain (PDB ID: 5b2c), in the monomeric form, were used for docking calculations. Missing hydrogen atoms were added, and protonation state of ionizable groups was computed by using Maestro Protein Preparation Wizard^51^. The structure was submitted to 100 000 steps of steepest descent minimization with MacroModel and optimized with OPLS_2005 force field, before being used for docking calculations. The 3D coordinates of substrates **1** and **2** were built with the help of Glycan builder^52^. In order to investigate the conformational behavior of the ligands, the three conformers (−*g*, +*g*, *g*) of α (2–3) Sia- Gal β (1–4)- NAcGlc were considered for docking purposes. The bonds were parametrized and Kollman charges added by means of Maestro. Then, the geometries of each ligands were optimized by 100000 step of steepest descent minimization with OPLS_2005 force field by using Macro Model^53^. Ligands were prepared for docking calculations using AutoDockTools^54^, setting all rotatable bonds free to move, except for the glycosidic linkages, during the docking calculations. Docking calculations of all compounds were performed by using AutoDock 4.2.2^55^. Analysis of the docking poses was performed with AutoDockTools. The docking protocol was validated by carrying out the docking of MuV-HN structure in complex with 3′ sialyllactose ligand (PDB-ID: 5b2d). The 3D structure of 3′ sialyllactose was extracted from the PDB file. The grid point spacing was set at 0.375 Ǻ, and a hexahedral box was built with x, y, z dimensions: 58 Ǻ, 56 Ǻ, 60 Ǻ centered in the centroid position among the active site residues Glu407, Arg422, Arg512, Tyr540, Glu561 and Asp204. A total of 200 runs using Lamarckian Genetic algorithm was performed, with a population size of 100 and 250000 energy evaluations.

### CORCEMA-ST calculations

The protons saturation of the ligand was predicted using CORCEMA-ST program and subsequently compared to the corresponding experimental results through the NOE R factor. The theory and the detailed of executing the CORCEMA-ST protocol have been previously described in detail^33^.

The pdb coordinates of the analyzed complexes were generated from docking calculations. The representative structures arising from the three possible ligand **1** conformers were selected from the cluster exhibiting lower energy and higher population and then submitted to the CORCEMA-ST protocol. The input parameters employed for the calculations according to the experimental conditions were saturation time of 2 s, protein concentration of 14 μM -HN and ligand concentration of 500 μM. A generalized order parameter S2 of 0.85 and a uniform leakage relaxation of 0.30 s^−1^ were postulated. As the dissociation constant (KD) of MuV-HN in complex with ligand has not been yet determined, the value was set in the range of 10^−4^–10^−6^ M and modified further to achieve the best fit. The direct saturation of the aromatic residues His, Trp, Phe and Thr protons was assumed, as the STD irradiation frequency was 6.5 ppm. The conformation of the ligand was considered to be the same in both free and bound state and the MuV-HN head domain in the monomeric form of 56 kDa. The k_on_ was set to 10^−8^ L mol^−1^ s^−1^, in the assumption of a diffusion controlled kinetic model; a correlation time, τ_r_, of the ligand in the free and bound state was set at 2 ns and 3 * 10^−8^ s was estimated through an empirical approximation^56^.

### Construction of expression plasmids, protein expression and purification

The DNA fragments encoding HN proteins (amino acid positions 96–582) were amplified by PCR from the template plasmids of the MuV Hoshino strain or the SBL-1 strain. They were individually cloned into the expression vector pHLsec containing the N-terminal secretion signal sequence and the C-terminal His_6_-tag sequence^54^. These expression plasmids were transiently transfected into 80% confluent HEK293S cells lacking *N*-acetylglucosaminyltransferase I [293S GnTI(-) cells]^57^ using polyethyleneimine-MAX (Polysciences) and incubated at 37 °C and 5% CO_2_. At 6 d post-transfection, the supernatant containing the secreted MuV-HN was harvested and centrifuged to eliminate cell components. The supernatant was incubated at 4 °C overnight and filtered to further eliminate insoluble components. MuV-HNs were purified using Ni^2+^-NTA affinity column (COSMOGEL His-Accept; Nacalai Tesque) in purification buffer (50 mM NaH_2_PO_4_, 150 mM NaCl, 10 mM imidazole, pH 8.0) and then eluted with elution buffer (50 mM NaH_2_PO_4_, 150 mM NaCl, 500 mM imidazole, pH 8.0). The eluted MuV-HNs were further purified using a size exclusion column (Superdex 200 Increase GL 10/300; GE Healthcare) in PBS without potassium. MuV-HNs were concentrated and adjusted to 1.5 mg/mL using Amicon Ultra centrifugal filters (Merck Millipore). Prior to perform NMR experiments, the buffer was exchanged to deuterated PBS by using vivaspin centrifugal filters and the final concentration of the protein was conveniently adjusted.

## Supplementary information

Supplementary Information.

## References

[1] Latner DR, Hickman CJ (2015). Remembering Mumps. PLoS Pathog..

[2] Ninth Report of the International Committee on Taxonomy of Viruses (Eds.: King, A. M. Q., Adams, M. J., Carstens, E. B. & Lefkowitz, E. J.), Elsevier, New York, 672–685 (2012).

[3] Rubin S, Eckhaus M, Rennick LJ, Bamford CG, Duprex WP (2015). Molecular biology, pathogenesis and pathology of mumps virus. J. Pathol..

[4] World Health Organization (2012). Mumps virus nomenclature update: 2012. Wkly Epidemiol. Rec..

[5] Rubin, S. A., Sauder, C. J. & Carbone, K. M. In Fi*eld* Virolo*gy* (Eds.: Knipe, D. M. & Howley, P. M.), Lippincott Williams & Wilkins, Philadelphia, 6th Ed, Vol I, 1024–1041 (2013).

[6] Cui A (2017). Mumps Epidemiology and Mumps Virus Genotypes Circulating in Mainland China during 2013–2015. PLoS One.

[7] Paterson, R. G. & Lamb, R. A. RNA editing by G-nucleotide insertion in mumps virus P-gene mRNA transcripts. *J. Virol.* **64**, 4137–4145, PMID: 2166809; PMCID: PMC247877 (1990). 10.1128/jvi.64.9.4137-4145.1990 PMC247877 2166809

[8] Elliott GD (1990). Strain-variable editing during transcription of the P gene of mumps virus may lead to the generation of non-structural proteins NS1 (V) and NS2. J. Gen. Virol..

[9] Wilson RL (2006). Function of small hydrophobic proteins of paramyxovirus. J. Virol..

[10] Elango N, Varsanyi TM, Ko$ vamees J, Norrby E (1988). Molecular cloning and characterization of six genes, determination of gene order and intergenic sequences and leader sequence of mumps virus. J. Gen. Virol..

[11] Elliott GD, Afzal MA, Martin SJ, Rima BK (1989). Nucleotide sequence of the matrix, fusion and putative SH protein genes of mumps virus and their deduced amino acid sequences. Virus Res..

[12] Lamb, R. A. & Parks, G. D. In Fiel*d V*irolo*gy* (Eds.: D. M. Knipe, P. M. Howley), Lippincott Williams & Wilkins, Philadelphia, 6th Ed, Vol I, 957–995 (2013).

[13] Orvell C, Alsheikhly A, Kalantari MB, Johansson B (1997). Characterization of genotype-specific epitopes of the HN protein of mumps virus. J. Gen. Virol..

[14] Lamb RA (1993). Paramyxovirus fusion: a hypothesis for changes. Virology.

[15] Schauer R, Johannis P, Kamerling JP (2018). Exploration of the Sialic Acid World. Adv Carbohydr Chem Biochem..

[16] Stehle T, Khan ZM (2014). Rules and exceptions: sialic acid variants and their role in determining viral tropism. J. Virol..

[17] Crennell S, Takimoto T, Portner A, Taylor G (2000). Crystal structure of the multifunctional paramyxovirus hemagglutinin-neuraminidase. Nat. Struct. Biol..

[18] Kubota M (2016). Trisaccharide containing α2,3-linked sialic acid is a receptor for mumps virus. Proc. Natl. Acad. Sci. USA.

[19] Yuan P (2005). Structural Studies of the Parainfluenza Virus 5 Hemagglutinin-Neuraminidase Tetramer in Complex with Its Receptor, Sialyllactose. Plos Path..

[20] Kubota M (2019). Molecular Mechanism of the Flexible Glycan Receptor Recognition by Mumps Virus. J. Virol..

[21] Takimoto T, Taylor GL, Connaris HC, Crennell SJ (2002). Portner, Role of the Hemagglutinin-Neuraminidase Protein in the Mechanism of Paramyxovirus-Cell Membrane Fusion. J. Virol..

[22] Kötzler MP, Blank S, Bantleon FI, Spillner E, Meyer B (2012). Donor substrate binding and enzymatic mechanism of human core α1,6-fucosyltransferase (FUT8). Biochim. Biophys. Acta.

[23] McCarter JD, Withers SG (1994). Mechanisms of enzymatic glycoside hydrolysis. Curr. Opin. Struct. Biol..

[24] Koshland D (1953). Stereochemistry and the mechanism of enzymatic reactions. Biol. Rev..

[25] Exnowitz F, Meyer B, Hackl T (2012). NMR for direct determination of K(m) and V(max) of enzyme reactions based on the Lambert W function-analysis of progress curves. Biochim. Biophys. Acta.

[26] Goličnik M (2011). Evaluation of enzyme kinetic parameters using explicit analytic approximations to the solution of the Michaelis–Menten equation. Biochem. Eng. J..

[27] Goličnik M (2010). Explicit reformulations of time-dependent solution for a Michaelis-Menten enzyme reaction model. Anal. Biochem..

[28] Voet, D. Voet, J. & Pratt, J. C. *Principles of Biochemistry*, Wiley, ISBN: 978-1-118-09244-6 (2008).

[29] Angulo J, Nieto PM (2011). STD-NMR: application to transient interactions between biomolecules—a quantitative approach. Eur. Biophys. J..

[30] Meyer B, Peters T (2003). NMR Spectroscopy Techniques for Screening and Identifying Ligand Binding to Protein Receptors. Angew. Chem., Int. Ed. Engl..

[31] Marchetti R (2016). “Rules of Engagement” of Protein–Glycoconjugate Interactions: A Molecular View Achievable by using NMR Spectroscopy and Molecular Modeling. ChemistryOpen.

[32] Marchetti R (2013). ChemBioChem.

[33] Poppe L, Brown GS, Philo JS, Nikrad PV, Shah BH (1997). Conformation of sLex Tetrasaccharide, Free in Solution and Bound to E-, P-, and L-Selectin. J. Am. Chem. Soc..

[34] Jayalakshmi V, Krishna NR (2002). Complete Relaxation and Conformational Exchange Matrix (CORCEMA) Analysis of Intermolecular Saturation Transfer Effects in Reversibly Forming Ligand–Receptor Complexes. J. Magn. Reson..

[35] Wen X, Yuan Y, Kuntz DA, Rose DR, Pinto BM (2005). A Combined STD-NMR/Molecular Modeling Protocol for Predicting the Binding Modes of the Glycosidase Inhibitors Kifunensine and Salacinol to Golgi α-Mannosidase II. Biochemistry.

[36] Schrodinger. Prime version 3.1, Schrodinger, LLC, New York, NY (2012).

[37] Schrodinger. Epik version 2.3, Schrodinger, LLC, New York, NY (2012).

[38] McAuley JL, Gilbertson BP, Trifkovic S, Brown LE, McKimm-Breschkin JL (2019). Influenza Virus Neuraminidase Structure and Functions. Front. Microbiol..

[39] Galazka, A. M., Robertson, S. E. & Kraigher, A. Mumps and mumps vaccine: a global review. *Bull. World Health Organ.* **77**(3–14), 10063655, https://apps.who.int/iris/handle/10665/56075 (1999). PMC2557572 10063655

[40] Rubin SA (2012). Recent mumps outbreaks in vaccinated populations: no evidence of immune escape. J Virol..

[41] Yoshida N (2008). Mumps virus reinfection is not a rare event confirmed by reverse transcription loop-mediated isothermal amplification. J. Med. Virol..

[42] Savage E (2005). Mumps outbreaks across England and Wales in 2004: observational study. Br. Med. J..

[43] Rydbeck R, Löve A, Örvell C, Norrby EJ (1986). Antigenic variation of envelope and internal proteins of mumps virus strains detected with monoclonal antibodies. Gen. Virol..

[44] Sauder CJ (2006). Changes in mumps virus neurovirulence phenotype associated with quasispecies heterogeneity. Virology.

[45] Peltola H (2007). Mumps outbreaks in Canada and the United States: time for new thinking on mumps vaccines. Clin. Infect. Dis..

[46] Šantak M (2013). Antigenic differences between vaccine and circulating wild-type mumps viruses decreases neutralization capacity of vaccine-induced antibodies. Epidemiol. Infect..

[47] Homan EJ, Bremel RD (2014). Are cases of mumps in vaccinated patients attributable to mismatches in both vaccine T-cell and B-cell epitopes?: An immunoinformatic analysis. Hum. Vaccin. Immunother..

[48] Schauer R (1982). *Chemistry, metabolism, and biological functions of sialic acids*. Adv. Carbohydr. Chem. Biochem..

[49] Kajihara Y (2004). Prompt chemoenzymatic synthesis of diverse complex-type oligosaccharides and its application to the solid-phase synthesis of a glycopeptide with Asn-linked sialyl-undeca- and asialo-nonasaccharides. Chem. Eur. J..

[50] Her C, Alonzo AP, Bang JY, Torres E, Krishnan VV (2015). Real-Time Enzyme Kinetics by Quantitative NMR Spectroscopy and Determination of the Michaelis–Menten Constant Using the Lambert-W Function. J. Chem. Educ..

[51] Schrödinger Suite 2019-2 Protein Preparation Wizard; Epik, Impact, Prime, Schrödinger, LLC, New York, NY (2019).

[52] http://glycan-builder.cermav.cnrs.fr/;

[53] MacroModel, Schrödinger, LLC, New York, NY (2019).

[54] Aricescu AR (2006). Eukaryotic expression: developments for structural proteomics. Acta Crystallogr. D. Biol. Crystallogr..

[55] Morris GM (2009). Autodock4 and AutoDockTools4: automated docking with selective receptor flexibility. J. Comput. Chem..

[56] Cantor, C. R. & Schimmel, P. R. Part II. Techniques for the study of biological structure and function. *Biophysical Chemistry* W. H. Freeman, Oxford, 503; 10.1016/0307-4412(81)90143-6 (1980).

[57] Reeves, P. J., Callewaert, N., Contreras, R. & Khorana H. G. Structure and function in rhodopsin: high-level expression of rhodopsin with restricted and homogeneous N-glycosylation by a tetracycline-inducible N-acetylglucosaminyltransferase I-negative HEK293S stable mammalian cell line. *Proc. Natl. Acad. Sci. USA* **15**, 99, 21, 13419–24; 10.1073/pnas.212519299 (2002). 10.1073/pnas.212519299 PMC129688 12370423

[58] Ohrui H, Nishida Y, Itoh H, Meguro H (1991). Preferred Conformation about the C5-C6 Bond of JV-Acetylneuraminyl(2-6)-D-galacto- and -D-glucopyranosides in Solution. J. Org. Chem..

