# Supplementary material for: Structural basis for Glycan-receptor binding by mumps virus hemagglutinin-neuraminidase
Source: Sci Rep. 2020 Jan 31;10:1589. doi: 10.1038/s41598-020-58559-6 (PMC6994497; doi:10.1038/s41598-020-58559-6)
Supplement: Supplementary file 1 — Supplementary Information. [file 41598_2020_58559_MOESM1_ESM.pdf]

Supplementary information

Structural basis for Glycan-receptor binding and its hydrolysis reaction by mumps virus hemagglutinin-neuraminidase

Rosa Ester Forgione[⊥a] and Cristina Di Carluccio[⊥a], Marie Kubota[b], Koichi Fukase[c], Yoshiyuki Manabe[c], Antonio Molinaro [a,c], Takao Hashiguchi[b], Roberta Marchetti[* a], Alba Silipo[* a]

Scheme S1 Substrates used in this study, representative of glycans exposed on glycolipids and glycoproteins of host cell surface.

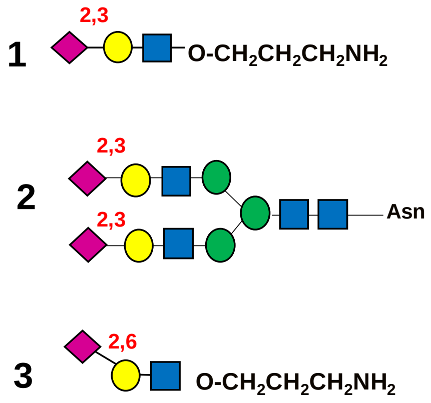

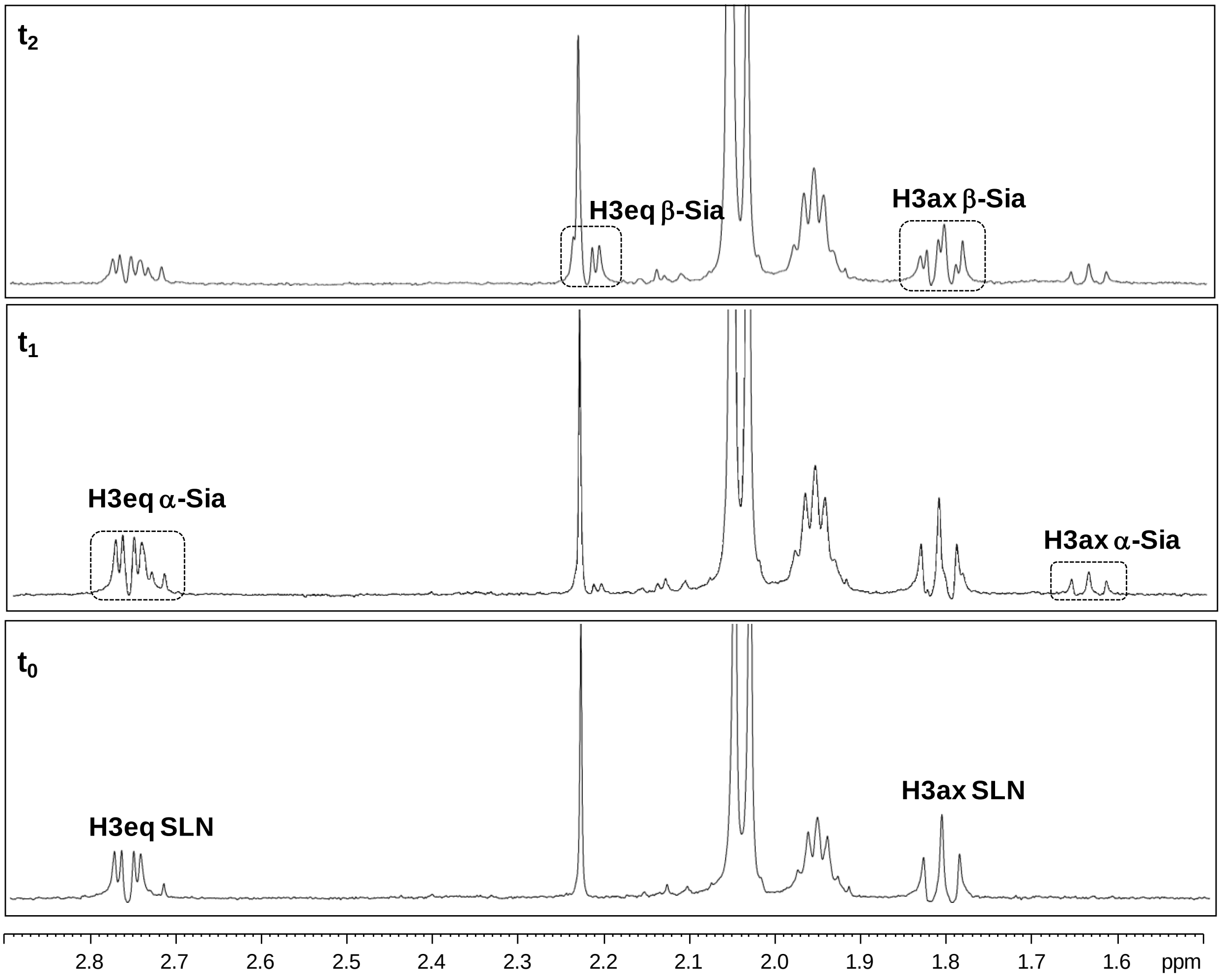

Figure S1 HN-MuV follows a retaining mechanism.

^{1}H NMR analysis shows the production of α-Neu5Ac after incubation of MuV-HN with substrate **1**, demonstrating that MuV-HN follows a retaining mechanism. Indeed, MuV-HN initially produces α-Neu5Ac from 3' sialylactosamine; the α-Neu5Ac undergoes mutarotation to form the more stable anomer, β-Neu5Ac, over time.

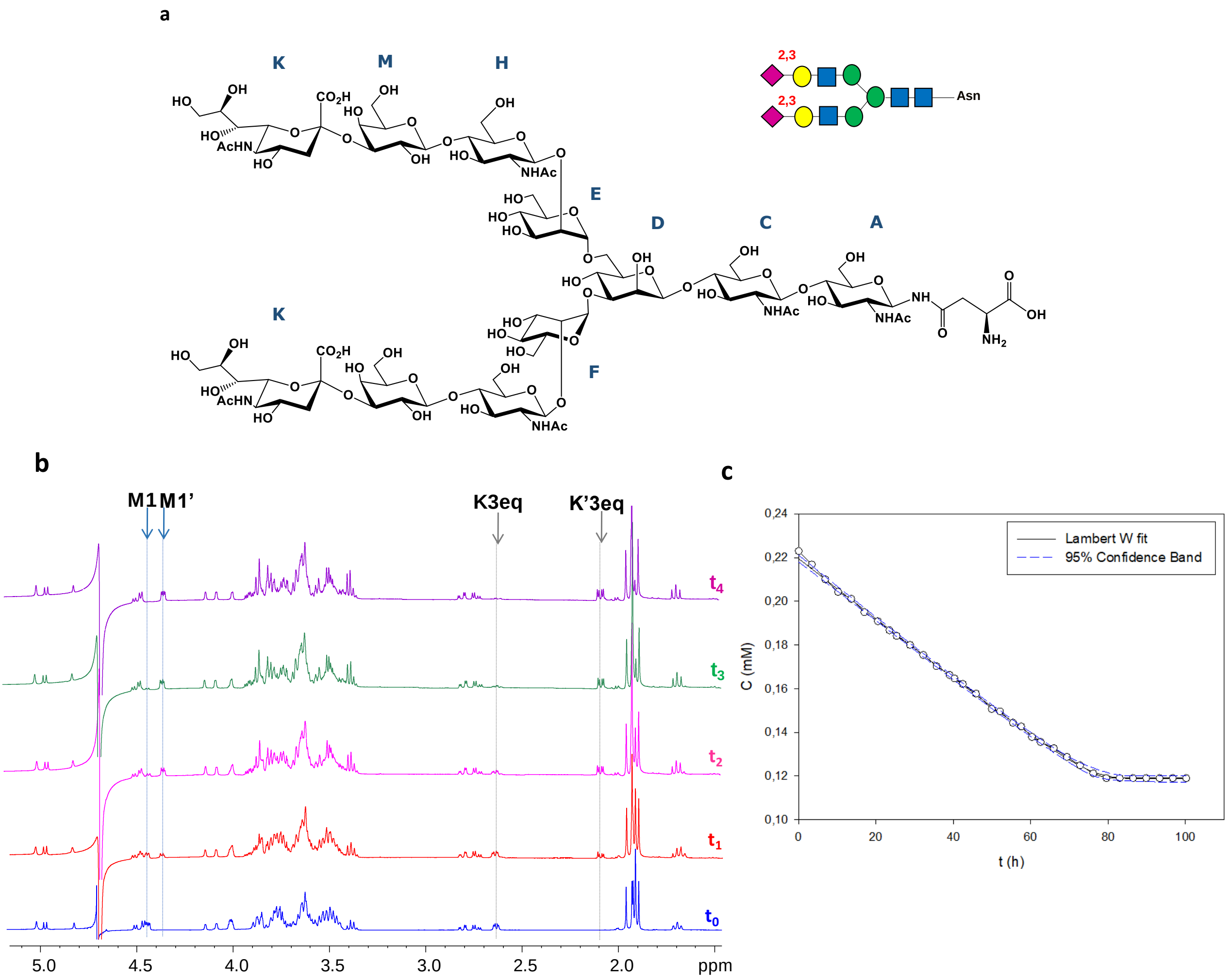

Figure S2. Kinetic analysis of the hydrolysis of undecasaccharide 2 by MuV-HN. **a** Structure of the sialylated undecasaccharide **2** studied in the interaction with MuV-HN is reported by using the 3D Symbol Nomenclature For Glycans (SNFG). Chem Draw 2006 Software (http://www.cambridgesoft.com) was used to draw the undecasaccharide. **b**. Section of ^{1}H-NMR spectra at different time of the enzymatic reaction of substrate **2** (T=298K, pH=7). The NMR quantification of the substrate concentration was performed by integration of the well-dispersed resonances of **1** (M1, K3eq). **b)** Analysis of the MuV-HN kinetics toward **2** by means of the Lambert W function. The fit of the kinetic data by the Lambert-W fit afforded a Km value of 3.5 µM and Vmax of $2.3*10^{-5}$ (mM/min). The blue dashed line represented the confidence interval of the fit.

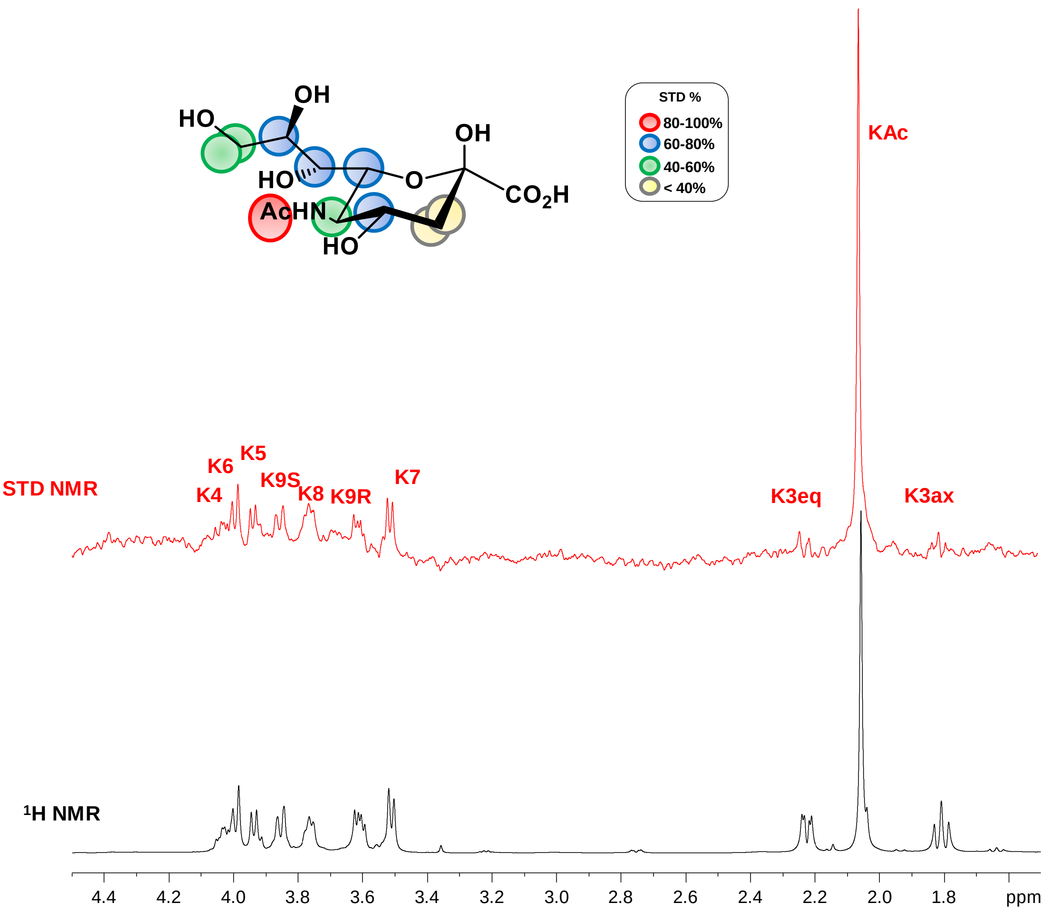

Figure S3 STD NMR analysis of reducing Sia in the interaction with by MuV-HN. a) The off-resonance spectrum as reference (black) and the STD (red) of MuV-HN/**1** mixture 1:70 at 283K. **b)** Epitope map of reducing Sia. STD percentages were calculated by $(I_0-I_{sat})/ I_0$ ratio, normalized with respect to the highest STD signal of the acetyl group belonging to sialic acid.

STD-NMR analysis of Neu5Ac-α(2,6)-Galβ(1,4)GalNAc (2) interacting with MuV-HN

STD NMR analysis was conducted to investigate the interaction between MuV-HN and the substrate **3** containing the α-2,6-linked sialic acid. In accordance with previously published fluorescence analysis data on the binding of MuV-HN to different types of α-2,6-linked sialyl glycopolymers, no STD NMR effects were observed for Muv-HN : **3** mixture (Figure S4), confirming that the receptor protein is able to specifically recognize and hydrolyse a-2,3 linked sialic acid.

Neu5Ac-α-(2,6)-Gal-β-(1,4)-GlcNAc-β-OR

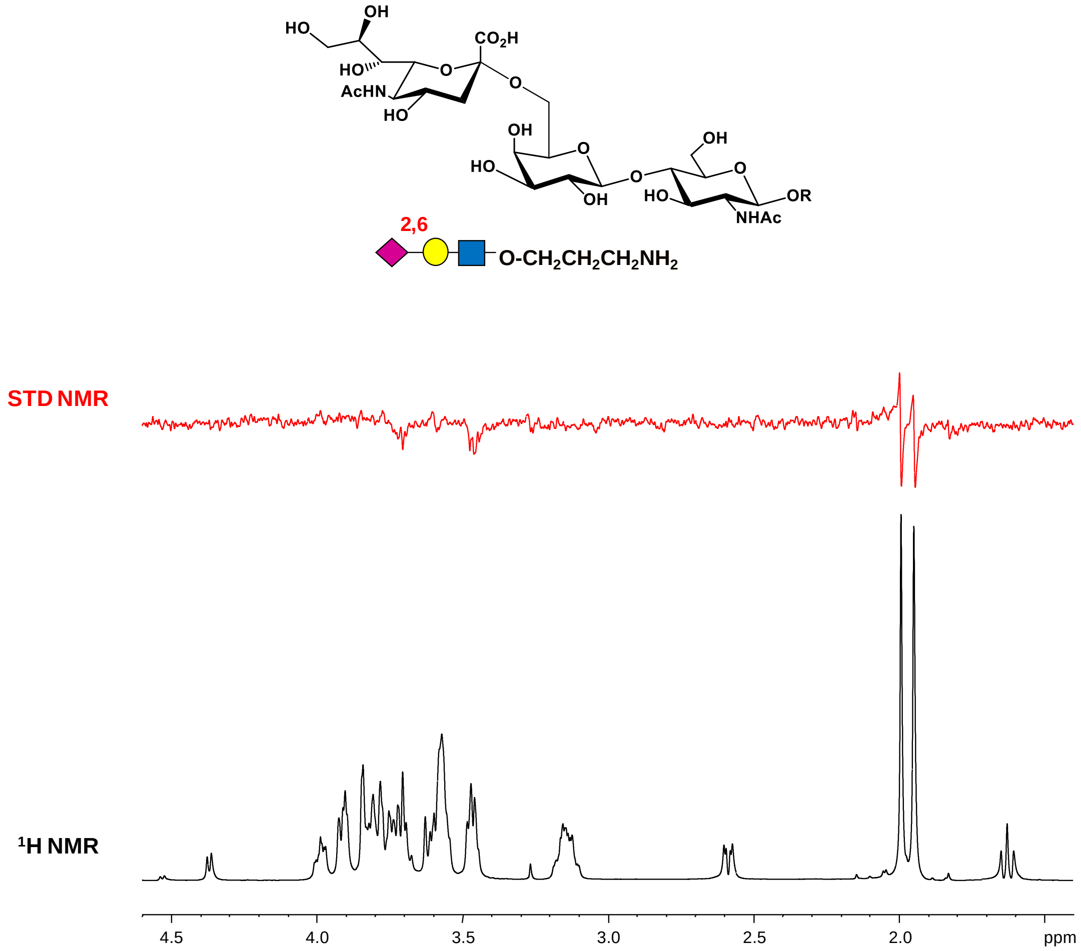

Figure S4. a Structure of the Neu5Ac-α(2,6)-Galβ(1,4)-GalNAc (trisaccharide **3**) studied in the interaction with MuV-HN is reported. The 3D Symbol Nomenclature For Glycans (SNFG) has been used. **b** STD NMR experiment of trisaccharide **3** in the interaction with MuV-HN at 283K.

a

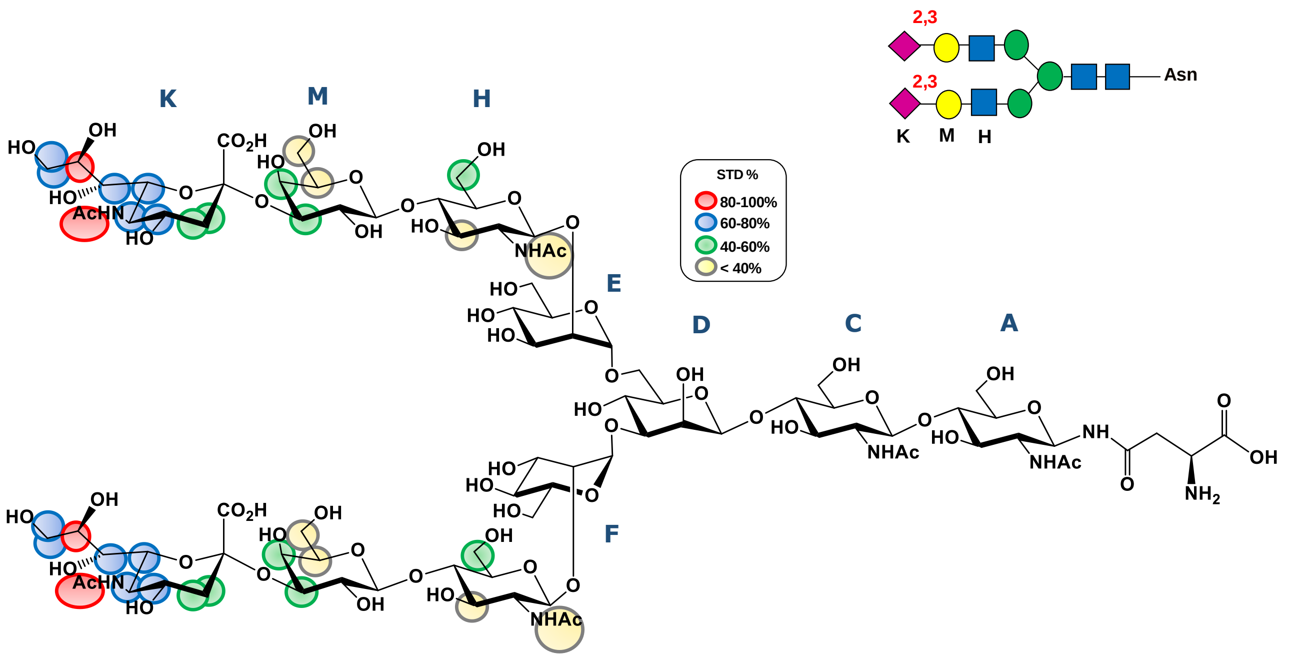

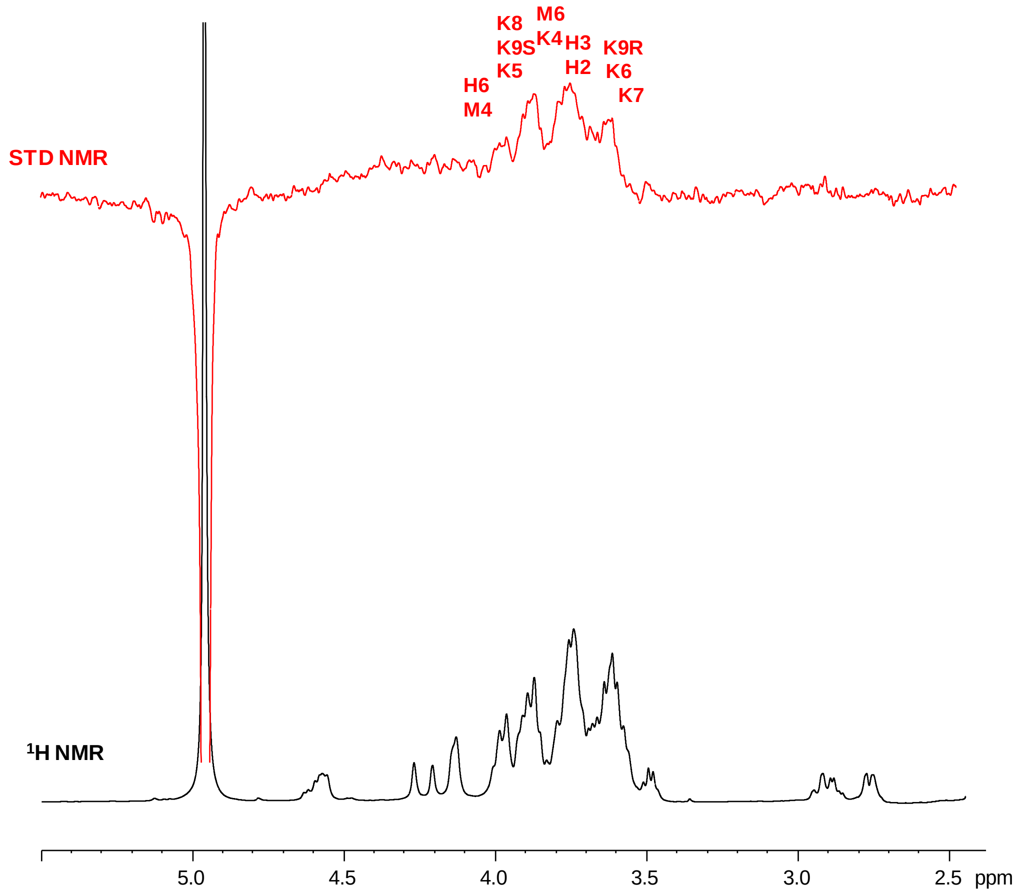

Figure S5. a) STD-derived Epitope mapping of undecasaccharide **2.** Chem Draw 2006 Software (http://www.cambridgesoft.com) was used to draw the undecasaccharide. **b)** STD NMR experiment of **2** in the interaction with MuV-HN at 283K.

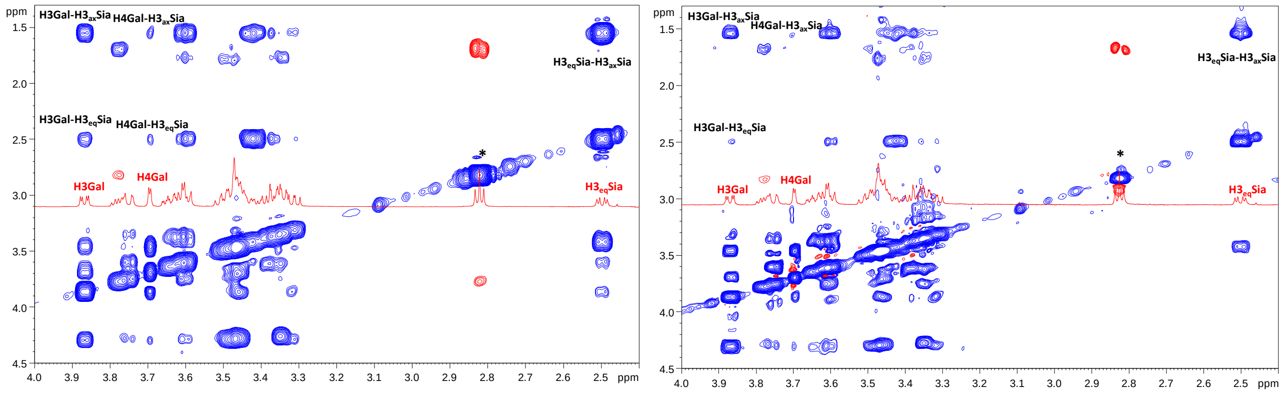

Figure S6 NOESY and tr-NOESY analysis of substrate 2. NOESY spectrum (**a**) of **1** in its free state (mixing time of 600ms) and tr-NOESY spectrum (**b**) of **1** bound to Muv-HN (mixing time of 400ms) at 283K. The protein-ligand molar ration was set at 1:30. Some differences in terms of signal intensities were observed comparing the free and bound state. The signal at 2.8 ppm marked with the asterisk belongs to the aglycon moiety at reducing end of **1**, which exhibits a high degree of flexibility.

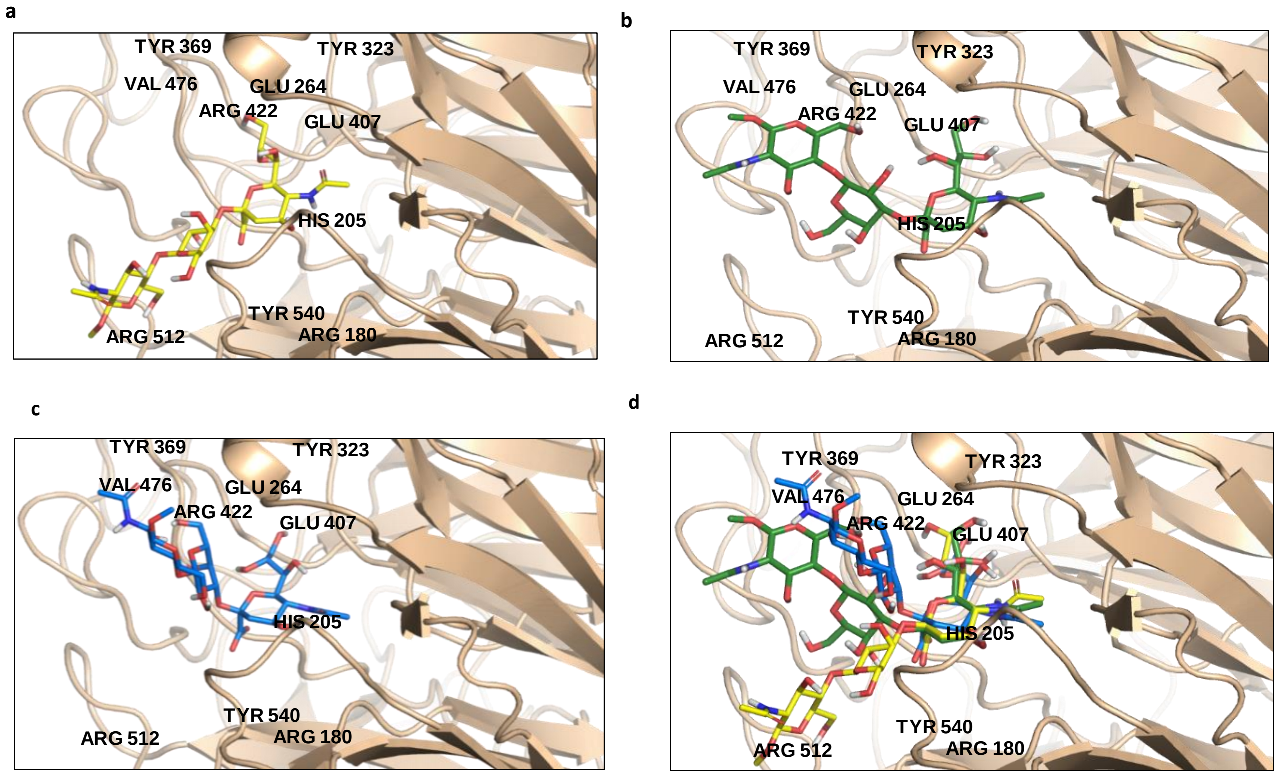

Figure S7. 3D model of MuV-HN in complex with substrate 1 derived by docking calculations a-d) Binding pocket of MuV-HN in the presence of the substrate **1** in the *g*, *-g* and *t* conformation, as derived by Autodock program. Only the amino-acid residues involved in the binding process are depicted. Green dotted lines stand for *inter*-molecular hydrogen bonds. Pymol 2.3 Software (https://pymol.org/2/) was used to draw the figure.

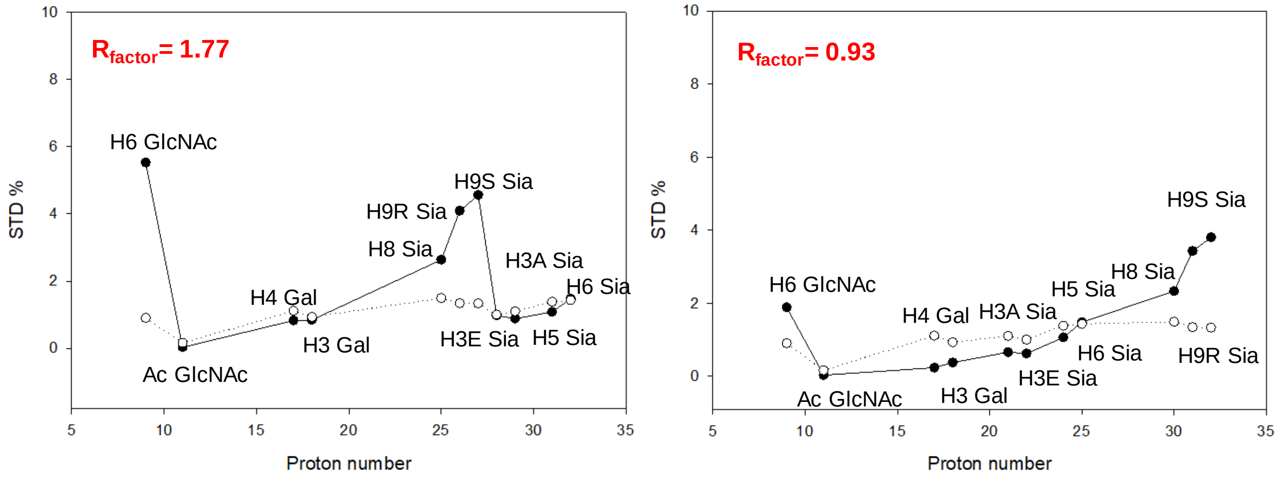

Figure S8. CORCEMA-ST analysis of other 3-D complexes obtained by docking calculations using different conformers. **a)** -g conformer **b)** g conformer.

Supporting Tables

Table S1 Docking results for the three possible conformers of substrate **1** obtained by using AutoDock 4.2.2. software

Conformer	Cluster rank	Number of conformations in this cluster	Number of conformations in this cluster (Å)	Estimated Free Energy of Binding (kcal/mol)	Estimated Inhibition Constant, Ki (mM)
t	1	124	55.996	-3.71	1.91 mM
g	1	104	56.729	-3.52	2.63 mM
-g	2	98	57.457	-2.75	9.72 mM
